# Tuberculose du médio-pied, une localisation inhabituelle: à propos d’un cas

**Published:** 2012-03-21

**Authors:** Hicham Hachimi, Latifa Tahiri, Nadira Kadi, Abdelhalim Ibrahimi, Abdelmajid Elmrini, Taoufik Harzy

**Affiliations:** 1 Service de rhumatologie CHU Hassan II, Fès, Maroc; 2 Service de traumatologie-orthopédie CHU Hassan II, Fès, Maroc

**Keywords:** Tuberculose osseuse, pied, cheville, lésion, bacille de Koch, ostéoarthrite, Maroc

## Abstract

La tuberculose ostéoarticulaire représente 2 à 5% de l’ensemble des tuberculoses, la localisation au médio-pieds est rare. Nous en rapportons le cas d’une patiente âgée de 18 ans, qui présente depuis 2 ans des douleurs du médio-pied droit inflammatoires avec tuméfactio, La radiographie a montré des géodes et des érosions des os naviculaire et cunéiformes. L’IRM a orienté vers le dignostic de tuberculose confirm par biospie et étude anathomopathologique. C’est pourquoi devant tout tableau clinique traînant ou devant toute lésion osseuse suspecte ou de présentation atypique le diagnostic de tuberculose doit être évoqué afin d’éviter un retard diagnostique.

## Introduction

La tuberculose constitue un problème de santé publique majeur dans le monde. Elle est responsable de trois millions de décès par année dont 95% enregistrés dans les pays du tiers monde. La tuberculose ostéo-articulaire représente 2 à 5% de l’ensemble des tuberculoses et 11 à 15% des tuberculoses extra-pulmonaires, la localisation au médio-pieds est rare. Nous en rapportons une nouvelle observation.

## Patient et observation

**Figure 1:**
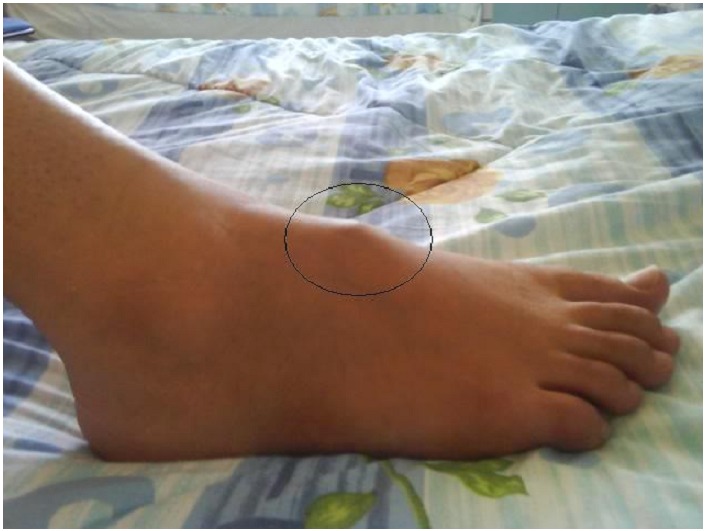
Synovite sans signe inflammatoire du médio-pied droit

**Figure 2:**
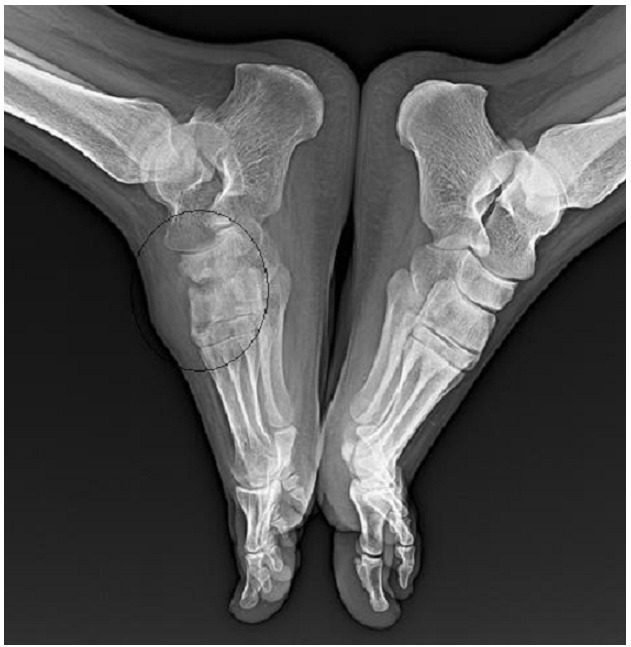
Géodes et érosions osseuses au niveau du médio-pied droit

**Figure 3:**
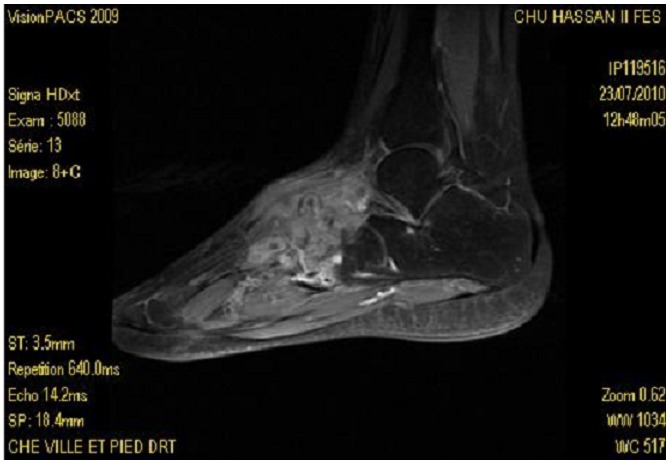
Coupe IRM sagittale des synovites des articulations du médio-pied avec larges géodes osseuses

Mlle K.K âgée de 18 ans, sans antécédents pathologiques notables, qui présente depuis 2 ans des douleurs de la cheville et du médio-pied droit d’horaire inflammatoire, quelques mois plus tard, ces douleurs se sont aggravées avec apparition de tuméfaction en regard du medio-pied droit sans signes inflammatoires en regard (Figure 1). Le tout évoluant dans un contexte d’apyrexie et de conservation de l’état général. L’examen clinique a objectivé la présence de synovite au niveau du médio-pieds sans signes inflammatoires en regard, la mobilisation des articulations du médio-pied était douloureuse. La patiente a bénéficiée d’un bilan biologique qui n’a pas montré de syndrome inflammatoire avec vitesse de sédimentation à 10 mm, et une C-réactive protéine à 7 mg/l, vu le caractère insidieux un bilan phtysiologique a était demandé, notamment une intradermoréaction à la tuberculine qui est revenue positive à 9mm, la recherche de bacille de koch dans les expectorations était négative et la radiographie de thorax ne montrait pas de lésion radiologique, notamment pas de signe radiologique en faveur de la tuberculose. La radiographie des pieds a montrée des géodes et des érosions osseuses des os naviculaire et des cunéiformes avec destruction des surfaces articulaires (Figure 2).d’ou la réalisation d’une IRM qui a révélée une synovite des articulations du médio-pied droit responsable de larges géodes osseuses et d’anomalies du signal évoquant en une arthropathie infectieuse type tuberculose (Figure 3). Une ponction articulaire avait objectivée une formule panachée, 1500 éléments blanc par mm3 avec 40% de lymphocytes et la recherche de bacilles de koch était négative a l’examen direct. La biopsie synoviale et osseuse avec étude anatomopathologique qui a montrée la présence de larges foyers de nécrose caséeuse sur un fond lymphoplasmocytaire et de nombreux follicules épithélioides et giganto-cellulaires confirmant la tuberculose du médio-pied.

Un traitement par antibiothérapie antibacillaire pendant six mois, conformément au programme national marocain de lutte anti-tuberculeuse, a été démarré chez la patiente à base de l’association éthambutol, pyrazinamide, isoniazide et rifampicine pendant deux mois puis l’association pyrazinamide et rifampicine pendant quatre mois.

## Discussion

En dépit de l′attention relativement limitée accordée à la tuberculose, elle reste une des principales infections qui cause des décès dans le monde entier [1,2]. La tuberculose ostéo-articulaire représente 2% à 5% de l’ensemble des tuberculoses et 11% à 15% des tuberculoses extra-pulmonaires [3,4]. La principale localisation est la tuberculose vertébrale qui représente au moins environ 50% des cas [5–8]. La localisation au niveau du pied et de la cheville est plus rare [9,10].

Les manifestations cliniques aspécifiques expliquent un retard diagnostique pouvant aller de quelques semaines à plusieurs mois [11,12]. Les principales manifestations cliniques sont la douleur, la tuméfaction et l’impotence fonctionnelle. Les manifestations systémiques comme la fièvre, les sudations nocturnes, l’amaigrissement sont rares. À des stades tardifs, on peut observer une fistulisation sur la peau [13]. L’intradermoréaction est positive dans 90% des cas de patients immunocompétents mais sa négativité n’exclut pas le diagnostic [14]. La radiographie du thorax n’est pas d’une grande spécificité car plus de 80% des patients n’ont pas de tuberculose active concomitante [15].

La radiographie osseuse standard est aspécifique. Au début, la tuméfaction des tissus mous et la décalcification peuvent être présentes avant les signes tardifs de destruction osseuse [15]. L’IRM reste l’examen de choix, elle est sensible dés le début de l’infection et permet de distinguer les zones osseuses infectées des zones saines et de montrer l’extension aux tissus mous et aux articulations avoisinantes.

Cependant, ni les signes cliniques, biologiques ni les signes d’imagerie ne permettent de confirmer le diagnostic. C’est la biopsie avec analyse histologique et bactériologique qui reste indispensable [16]. Parfois le diagnostic de l’ostéoarthrite tuberculeuse est difficile, vu le caractère paucibacillaire, et la confirmation se fait par méthode PCR, cette technique se base sur la mise en évidence de l′ADN mycobactérien par la PCR avec une spécificité de 92% à 98% [17,18].

Le traitement est essentiellement médical par antibacillaire qui permet de limiter l’évolution vers les séquelles douloureux et les déformations, la majorité des lésions guérissent en 6-12 semaines sous le traitement médical.

Le traitement chirurgical est indiqué en cas d’insuffisance du traitement médical, lorsque persiste une synovite, une fistule ou un abcès. Enfin la chirurgie permet de corriger les déformations séquellaires et douloureuses [19,20]. C’est principalement les gestes d’arthrodèses qui sont indiqué au niveau du pied et de la cheville.

## Conclusion

L’atteinte ostéo-articulaire du pied par le bacille de Koch est rare, c’est pourquoi devant tout tableau clinique traînant ou devant toute lésion osseuse suspecte ou de présentation atypique le diagnostic de tuberculose doit être évoqué afin d’éviter un retard diagnostique et permettre une prise en charge thérapeutique précoce.
